# Toward Effective Monitoring of Diffuse VOC Emissions: A Critical Discussion and Review of the Applications of EN 17628:2022

**DOI:** 10.3390/s25051561

**Published:** 2025-03-03

**Authors:** Luca Carrera, Selena Sironi, Marzio Invernizzi

**Affiliations:** Department of Chemistry, Materials and Chemical Engineering “Giulio Natta”, Politecnico di Milano, Piazza Leonardo da Vinci 32, 20133 Milan, Italy; luca.carrera@polimi.it (L.C.); marzio.invernizzi@polimi.it (M.I.)

**Keywords:** diffuse VOC emissions, remote gas sensing, gas monitoring techniques, environmental monitoring, EN 17628:2022 standard

## Abstract

**Highlights:**

**What are the main findings?**

- EN 17628:2022 introduces a technical framework for monitoring diffuse VOC emissions, outlining five techniques for detection, localization, and quantification.
- The analysis highlights the strengths and limitations of the described methodologies in the characterization of complex emissions from industrial sites.

**What is the implication of the main finding?**

- An accurate selection of monitoring techniques is crucial for improving the reliability of emission flux estimates.
- The integration of complementary techniques enables a more robust analysis of emissions, addressing the complexity of diffuse sources in an industrial context

**Abstract:**

The estimation and characterization of diffuse emissions of volatile organic compounds (VOCs) is a crucial issue for industry and environmental regulators. Compared to channelled ones, diffuse emissions derive from complex (non-point) sources, such as wastewater treatment plants, storage tanks, and process unit components. Such sources are typically influenced by dynamic factors such as operational activities and weather conditions. Therefore, this complexity makes the localization and quantification of diffuse VOC emissions a crucial challenge from a technical and regulatory perspective. Recently, the technical standard EN 17628:2022 has been published, which provides a framework to address this issue, proposing five different techniques for the localization, identification, and quantification of diffuse emissions. Nevertheless, while it represents a step forward in this field, the standard shows some shortcomings for a proper implementation, potentially causing divergent interpretations of the guidelines. The accuracy of the measurements is highly dependent on the configuration and morphology of the site, but especially on the meteorological data implemented to calculate the emitted flux. In addition, these techniques, despite being well-established, are particularly complex from both a technical–scientific and logistical–economic point of view. An emerging method, Quantitative Optical Gas Imaging (QOGI) appears to theoretically overcome some issues, but requires further studies to ensure accurate and reproducible quantification of emissions. This review aims to highlight the advantages, disadvantages, and potential developments of the various techniques described in the standard for the characterization of diffuse VOC emissions in the industrial sector.

## 1. Introduction

Industrial emissions of volatile organic compounds (VOCs) represent a highly topical issue for environmental and health protection, as they contribute significantly to air pollution, worsening human well-being [1,2,3,4], and potentially causing odour impact due to their odour potential [5,6,7,8,9,10,11]. In detail, diffuse emissions, i.e., all those sources not defined by a specific emission point, are still complex to characterize and monitor, given the strong dependence on dynamic factors such as process operational activities and environmental conditions [12,13,14]. In fact, unlike point emissions, which are easier to identify and monitor, diffuse emissions come from nonspecific points, such as storage tanks [15,16,17,18], wastewater treatment basins [19,20,21], process unit components [22,23,24], or solid biomass piles [5,25,26]. Such sources emit intermittently and unpredictably, making their detection, quantification, and subsequent mitigation complicated [27,28].

Over the past decades, the absence of standardized approaches for the characterization of diffuse emissions of VOCs has led to inconsistencies in detection, limiting the development of robust techniques and strategies for the detection, quantification, and mitigation of diffuse emissions [29]. These difficulties are further compounded by the complexity of the available monitoring techniques, which, even if well-established, have strong dependencies on site layout and configuration, meteorological conditions, and expert interpretation [30]. The introduction of EN 17628:2022 [31] is therefore an important step forward in addressing this issue. This standard provides a comprehensive framework for the selection and application of the most appropriate technique to use depending on the objective of monitoring, be it the detection, identification, or quantification of diffuse VOC emissions [30]. The standard, developed in line with European regulatory requirements and already cited in industry Best Available Techniques (BAT) guidelines [10,32,33], aims to unify and promote available techniques, some of which are already applied in industrial settings.

EN 17628:2022, specifically, describes five techniques for monitoring diffuse sources, which are based on indirect methods for estimating emissions, encompassing optical methods, the use of tracer gases, and inverse simulation of atmospheric dispersion.

Alongside the methods described in the standard, so-called ‘micrometeorological’ methods are also available for indirectly estimating pollutant mass fluxes from complex sources [34,35,36]. However, these techniques are not mentioned in the cited reference.

This review provides a detailed analysis of the EN 17628:2022 standard, critically examining its content, its applicability, and potential areas of improvement of the listed methods, including a comparison of applicative case studies available in the literature. Each technique is explored in depth, outlining the operation principles, strengths, and technical limitations of these measurement methodologies. Based on case studies and empirical data drawn from the literature, this review aims to evaluate the effectiveness of these techniques, particularly the optical ones, in various industrial settings, shedding light on the operational challenges and effective operation of detection, identification, localization, and, most importantly, quantification systems.

This review aims to support industry, regulatory, and research stakeholders in optimizing VOC monitoring practices. In particular, it highlights the importance of an appropriate selection of the monitoring technique, balancing technical accuracy with feasibility.

## 2. General Overview and Selection of Monitoring Technique

### 2.1. General Overview of the Standard

The EN 17628:2022 standard [31] was developed to provide a framework for the selection and use of monitoring methods (detection, identification, and/or quantification) for the emission of VOCs into the air from diffuse sources in the industrial field. Specifically, it explicitly refers to certain types of emission sources, such as wastewater treatment tanks, storage tanks, and operational units of production plants, as well as to the phases of storage, transfer, and handling of these compounds.

This technical standard theoretically describes indirect methods for quantitative measurement of VOC flows from non-point sources. These measurements are included in the context of various BREF (Best Available Techniques Reference) documents and support the Best Available Techniques Conclusions [37] that require the evaluation and quantitative reporting of diffuse VOC emissions. At the time of writing of this document, the mentions of this technical standard are included in the following documents: Mineral Oil and Gas Refineries (BAT 6) [38], Common Waste Water and Waste Gas in the Chemical Sector (BAT 5) [33] and Common Waste Gas Management and Treatment Systems in the Chemical Sector (BAT 22) [32].

EN 17628:2022 was formulated in response to mandate M/514 (2012) [39] issued by the European Commission to standardization bodies in Europe. This mandate anticipated that the ongoing revisions of Best Available Techniques (BAT) conclusions for the refining [10] and chemical industries [33] would include provisions for the quantification of diffuse VOC emissions [30].

The methods described in the standard, useful for detecting and/or identifying and/or quantifying emissions, are the following five:

- Differential Infrared Absorption Lidar (DIAL)
- Solar Occultation Flux (SOF)
- Tracer Correlation (TC)
- Optical Gas Imaging (OGI)
- Reverse Dispersion Modelling (RDM)

In particular, the standard describes the fundamental principles on which each technique is based, as well as the technical requirements for use and existing limitations.

A peculiarity of the EN 17628:2022 standard [31] lies in the marked complementarity and alternativeness of the techniques presented for a complete characterization of the emissive scenario. For example, periodic leak detection and repair (LDAR) controls of process fittings (i.e., valves, flanges, or compressors) described in the EN 15446:2008 standard [40] can be performed using the OGI technique, described in the new document. Given the speed of using this new technique, compared to conventional LDAR, this approach is often referred to as *Smart LDAR* [41]. Larger-scale surveys (such as entire production sites or plant sections) can be carried out using DIAL and/or SOF techniques. Lastly, in cases where the emission source is known and already located, the TC and RDM techniques can be used to quantify the emission rate.

Before delving into the individual techniques and methods described in the standard, it is useful to spend a paragraph on two particular terms whose use is often confusing, to have a better understanding of the scope of application of the standard. Specifically, it is important to distinguish between the terms “*fugitive emission*” and “*diffuse emission*”:

- “*Fugitive emission, an emission to the atmosphere caused by loss of tightness of an item which is designed to be tight*” [31,40];
- “*Diffuse emission, an emission to the atmosphere from an identified site or facility, not specifically directed to identified stack emission points*” [31].

### 2.2. Monitoring Program and Technique Selection

A VOC monitoring program cannot neglect diffuse emissions, and it is characterized by the following three main objectives:

- The detection of each emission source
- Its localization
- The quantification of the emission rate

The first step in conducting this assessment is to create an inventory of the known emission sources at the site under consideration and, if possible, to collect data obtained from previous monitoring studies. Additionally, the markedly variable nature of these emissions, both in terms of source type and the temporal variability of the phenomenon, requires in-depth evaluations regarding the choice of the most appropriate monitoring method. The parameters to be considered are the following:

- Purpose and objective of the monitoring
- Type of monitoring required (identification and/or localization and/or quantification)
- Spatial resolution (function of the chosen technique and the area to be monitored)
- Localization of the emission source
- Quantification of the emission rate
- Duration of the monitoring (to consider both stationary sources and emissions due to malfunctions and/or emergencies)
- Chemical species to be monitored

**Figure 1:**
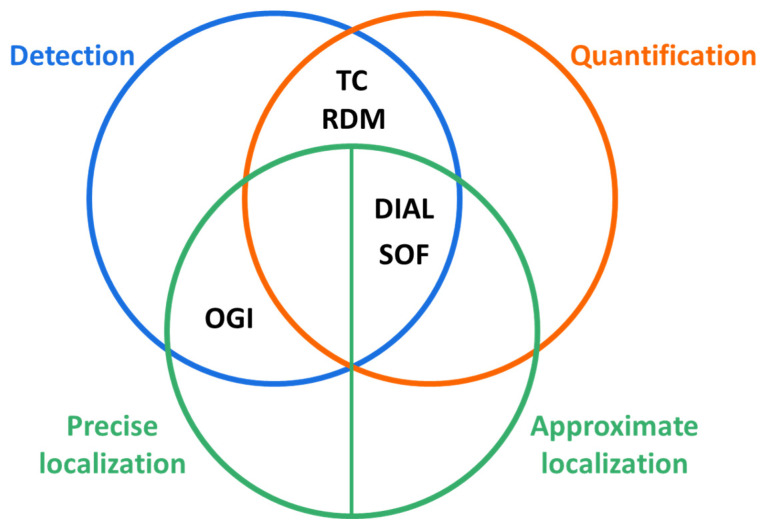
Applicability of the different monitoring techniques.

Following the preliminary assessment based on the parameters described above, Figure 1 can be used to evaluate which monitoring method is most suitable for the situation under examination.

A quick way to choose the most appropriate technique is to evaluate the size of the source to be monitored. For instance, OGI is the best method for assessing fugitive emissions from pumps, valves, or flanges since it enables the identification and accurate localization of the emission source. Conversely, for monitoring large-scale diffuse emissions (i.e., tank farms, water treatment sections, or entire industrial sites), the most suitable techniques appear to be DIAL and SOF, which allow for the detection and quantification of emission sources, but only approximate the localization of the single source itself. Additionally, the TC and RDM techniques, valid to detect and quantify the emission rate, also allow for the approximate localization of emission sources when used in conjunction with OGI, as clearly stated in the EN 17628:2022 standard in sections 8.1.2.4 and 8.1.2.5. Therefore, the complementary nature of the described techniques is evident when a very detailed understanding of the emission scenario is necessary.

Finally, before detailing the background of each technique, it is important to emphasize that all methods are highly dependent on external and uncontrolled variables, particularly weather conditions and the accessibility of potential emission points. Furthermore, the methods outlined in the standard are applicable for determining emissions within the monitoring period, but they are not suitable for extrapolating data to periods beyond the monitoring timeframe [30].

## 3. Discussion About the Monitoring Techniques Described in the Standard

### 3.1. Differential Infrared Absorption Lidar (DIAL)

The DIAL is a method that uses laser sources in the infrared (IR) and ultraviolet (UV) regions to detect and quantify emissions of specific VOCs and the corresponding flows directly in the atmosphere [42].

**Figure 2:**
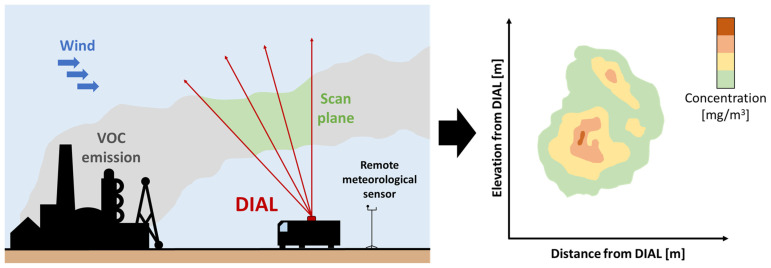
Operating principle of the DIAL technique.

This technique employs laser pulses transmitted through the air to determine the concentration and position of a target gas, allowing the detection of emissions over an aerial section of around 500–800 m, with a spatial resolution of approximately 3 m [43]. By combining this information with anemometric data (i.e., wind speed field), it is possible to calculate the emissive flows. The DIAL system, typically mounted on a mobile platform, being an active system, is capable of operating under various environmental conditions and is usually used to detect and quantify emissions from specific sections of an industrial site. The system is aimed at the detection, localization, and quantification of one or two species at a time and is generally not sensitive to emissions dispersed over long distances. An operational scheme of the DIAL technique is shown in Figure 2.

To monitor VOCs, particularly hydrocarbons, the DIAL must operate in the mid-IR region (2.5–5 μm), while for aromatic compounds, measurement in the UV region is required.

The selection of measurement areas depends on various factors, primarily the wind direction. Based on this, the most suitable measurement locations are identified from both a technical perspective (measurement field, upwind sources, …) and a logistical perspective (occupiable areas, potential obstacles, …). The first area where measurements should be taken is the one without contribution and upwind of the rest of the site under investigation. The idea is then to move in the direction of the wind to determine the upwind contribution for each measured area as the sum of the previously measured contributions. Additionally, identifying probable emission sources and the species involved allows for a better detection and quantification. Given the importance of meteorological data for quantitative estimation of flows in the described method, if the area’s topography is complex, the standard suggests installing additional meteorological stations in the vicinity of the measurement area.

During the measurements, it is essential to select the laser wavelengths so that the differential absorption is adequate and other atmospheric species do not interfere. The verification of any interference can be conducted by analyzing spectral databases, for example, those collected from the HITRAN [44] or NIST [45] databases.

The vertical DIAL scan, which consists of a series of measurement lines at different elevation angles, must be configured to correctly capture emissions and resolve the concentration as a function of distance along the line of sight.

The acquired data is then analyzed to obtain concentrations resolved along the optical pathway of each elevation angle. From the return signal, the path-integrated concentration of the target species is subsequently calculated. Polar concentrations are obtained and then combined to produce a Cartesian concentration profile. Finally, the wind speed profile is coupled with the two-dimensional mapping of the concentrations over the measured area, in order to estimate the related emission flux value.

Currently, in Europe, there is only one commercially available DIAL service for the quantification of diffuse emissions from large complexes. This facility is managed by the National Physical Laboratory (NPL) [46] of the United Kingdom and is capable of operating in both IR and UV spectral regions.

Numerous comparative tests have been conducted to validate the flow measurement obtained using DIAL from known emission sources or those directly measured at the source. For example, the NPL has performed measurements downstream of a known methane flow in open field and flat terrain conditions and achieved agreement within ±10% [47]. The NPL itself acknowledges that “*such tests mainly highlight the uncertainties in the technique concerning the calculation of emissive flows, which depend on an accurate definition of the wind speed profile*”. The accuracy of such profiles will be significantly worse within large industrial plants, as, given the topography of these areas, the actual wind profiles will be very different from those under ideal open fields and flat terrain conditions. Moreover, indications about the typical sensitivity values for a wide range of pollutants are reported [47].

Considering all the mentioned references, it is possible to highlight the advantages and disadvantages of the DIAL technique.

In particular, the following advantages can be listed:

1. DIAL is a well-established technique developed in the 1980s [48,49] and is still used commercially today.
2. The scanning system provides a two-dimensional concentration map in the air, allowing for the estimation of the plume’s shape and height. With a proper choice of scanning point, the technique can be used to visualize the specific area from which the plume extends, though it cannot pinpoint the exact emission source. For precise localization, a DIAL system may be complemented with an OGI thermographic inspection.
3. The technique can quantify the mass flow emitted during the scan period of the perpendicular wind speed field to the scan plane, which can be estimated. The primary errors in quantifying flows arise from the description of the wind field.
4. When positioned to perform a horizontal scan, the DIAL system can effectively identify significant emitters in the area (e.g., tank farms). However, the resolution (10 m) is not sufficient to identify individual significant point emitters.
5. The DIAL system can quantify emissions from specific challenging sources. For example, it can determine emissions from flaring combustion if the flows and compositions sent to the flare are known, thus enabling the measurement of the flare’s efficiency and the implementation of emission factor estimates.

On the other hand, regarding the disadvantages currently associated with the DIAL technique, the following can be stated:

1. The accuracy of emission rate measurements depends on weather conditions. Measurements cannot be conducted when visibility is significantly reduced due to fog or rain, or under very low wind conditions.
2. To ideally obtain emitted mass flows, each concentration measurement point must be multiplied by the perpendicular wind speed component at the same spatial point. This is impracticable because the wind field cannot be measured at the exact concentration measurement point. Additionally, the emission plume near the source may be in the building downwash zone, causing the wind profile to vary with distance and differ significantly from open field conditions. This can lead to significant overestimation of the emission flow.
3. Background concentrations upwind of the investigated source must be subtracted from downwind measurements. However, with only one DIAL system, it is not possible to simultaneously measure the upwind and downwind of the emission source. The DIAL setup involves large mobile containers (e.g., the NPL van is 12 m long), and moving and re-establishing these for upwind measurements takes considerable time (about an hour). During short-term monitoring campaigns, only one upstream scan might be feasible, making it impossible to determine if intermittent upstream sources influenced downwind measurements.
4. Single-source measurements are typically performed over short periods, in fact, five to ten scans provide 1 to 2 h of measurement. Emissions in facilities like petrochemical plants and refineries often vary temporally. Thus, short-term DIAL measurements can only provide a “*snapshot*” of the emission flow from these sources. While DIAL data can help identify significant emitters, extrapolating to provide long-term estimates can lead to significant errors.
5. Many hydrocarbon absorption spectra detected by DIAL overlap, and water vapor interference is certain. Operators often use the absorption frequency that gives strong signals for a typical hydrocarbon mixture in a refinery. The wavelength of a typical refinery hydrocarbon mixture spans from the visible to the mid-IR region, roughly between 0.5 and 2 μm [50]. This will cause systematic errors if the investigated pollutant has absorption characteristics significantly different from the standard hydrocarbon mixture (e.g., a mixture of propane and pentane when evaluating total hydrocarbons).
6. Currently, only one company in the world (NPL) offers the DIAL system for commercial purposes.
7. The system requires a team of no less than two highly prepared professionals. Typically, a measurement campaign at an industrial plant usually spans around 10 working days, with total costs > €10,000 per day.

### 3.2. Solar Occultation Flux (SOF)

The SOF method is employed to map and measure gas emissions across various scales, ranging from entire sites to individual equipment. This method efficiently analyzes large sections of a facility and helps identify the most significant emission sources. The SOF technique relies heavily on direct sunlight and utilizes spectrometry to analyze the sunlight radiation through the atmosphere using a mobile system [51].

The SOF system must be equipped with an IR spectrometer (Fourier Transform Infrared, FTIR) with a spectral resolution of 1 cm^−1^ in the spectral region between 3.2 μm and 12.5 μm and a high-precision solar tracker. This tracker is a mechanical automatic system that moves the detector to optimize sunlight exposure, similar to systems used to maximize the efficiency of photovoltaic panels by rotating them based on the sun’s position.

**Figure 3:**
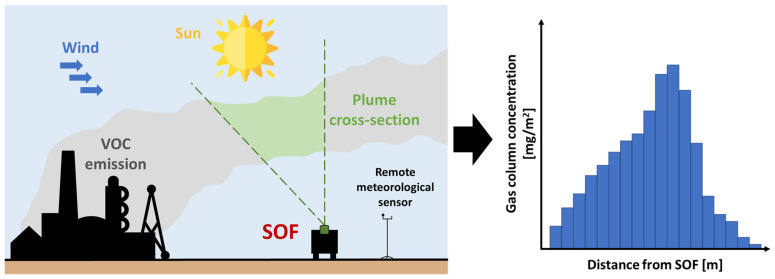
Operating principle of the SOF technique.

The mobile platform, equipped with sensors, moves transversely downwind relative to the emission source. This movement intersects the plume with the measurement path to obtain an integrated concentration profile. Measurements are typically conducted along the periphery of emission areas, allowing for the subtraction of the upwind component and combining the integrated concentration of respective paths with wind information to determine emission fluxes (Figure 3).

For practical applications on an industrial scale, especially for monitoring emissions of alkanes, alcohols, and alkenes, the SOF system must be securely mounted on a mobile platform, enabling direct solar infrared (IR-SOF) measurements. Before deployment, it is crucial to conduct a preliminary meteorological analysis using historical data to evaluate wind speed and direction ranges, cloud cover, and expected precipitation. The measurement plan may need to be adjusted based on wind direction changes throughout the day.

Simulating the absorption spectra of background species (e.g., H_2_O, CO_2_, and CH_4_) present in the atmosphere using data from the HITRAN [44] or NIST [45] databases is essential. Calibration spectra are then used to detect VOCs.

Quantifying column values, which are the integrated concentrations along the solar beam, is based on the Beer–Lambert law. According to this law, the logarithm of the ratio of the intensity of light absorbed by the gas, defined as absorbance (*A*), is proportional to the average path concentration, *C*.

(1) $$A=\log\left(\frac{I_{0}}{I}\right)=\varepsilon Cl$$

where *I*_0_ is defined as the incident light intensity, *I* is defined as the transmitted light intensity, *ε* is defined as the molar extinction coefficient (i.e., a characteristic parameter for each chemical species [52]), and *l* is the optical path length of the light.

Particular attention is required for alkanes since their absorption characteristics are similar and interfere with each other. However, since the number of absorbed C-H bonds is directly correlated with the molecular mass, the total mass of the alkane can be obtained with good accuracy despite the cross-interferences.

The SOF technique provides integral average values of concentration along the optical path; this means that it measures the total gas concentration over a given optical path without providing detailed information about the spatial distribution of the concentration along the path. Additionally, the EN 17628:2022 standard [31] asserts that the SOF system requires a detection limit of at least 10 mg/m^2^ for gases and continuous wind measurement at 10 m height with a temporal resolution of at least 1 min.

The mass flux along a measurement path is obtained as the product of the columns measured by the SOF integrated along the path, multiplied—also as in the DIAL case—by the wind vector component blowing orthogonal to the direction of the path.

On the other hand, SOF measurements have some limitations related to the need for the measurement vehicle to traverse roads both upwind and downwind of the sources, maintaining a clear line of sight to the sun. The SOF survey is therefore limited in its ability to separate emissions from roads and other obstacles, and individual measurements are temporally discrete and cannot be averaged or extrapolated over time.

The SOF is a relatively recent technique, developed towards the end of the 1990s [51,53,54], and has been used operationally to measure industrial emissions remotely since 2002. FluxSense AB [55], a Swedish company specialized in monitoring emissions from industrial plants and refineries, is currently the only commercial provider of this technique.

The measurement error for SOF is primarily due to uncertainties in wind data used for flux calculations. Given that, total emissions from industrial facilities are commonly measured at distances ranging from 0.5 km to 3 km from the sources, and the majority of the smoke plume has ascended several hundred meters by the time it crosses the scan line. In that case, the wind uncertainty is estimated by the provider FluxSense to be around 30% [56]. Measurements conducted within industrial areas, near emission zones, are associated with greater uncertainties (estimated by FluxSense to be up to 50%, but possibly higher depending on topography) due to increased complexity in the wind field. FluxSense indicates that other possible sources of error are the parameters of the absorption line of the detected compounds, with uncertainties of 3% for the spectroscopic part.

Multiple validation studies of the SOF technique have been carried out, using a tracer gas (SF_6_) that was released in known quantities and subsequently measured using the SOF method. In one experiment, SF_6_ was emitted from the top of a 17-meter-high pole in an open field. Downwind cross-sectional measurements were then performed with the SOF measurement system at various distances from the source. The average emission value detected differed by 11% from the actual value, but discrepancies of up to 50% were obtained for individual measurements. SF_6_ releases from oil tanks at various refineries were also conducted, showing discrepancies of 50% for close measurements, but more contained (around 30%) when measured at greater distances [57].

Considering the mentioned references, the advantages and disadvantages of the SOF technique can be highlighted. In particular, the following benefits can be reported:

1. Despite being a recent development, the technique has been used internationally in several projects and can be considered well established.
2. The quantification of emitted mass flux can be carried out if a reasonable estimate of the wind field can be determined during the measurement period. As with DIAL, the main error in determining the flux is introduced by wind field data.
3. The method is simpler than DIAL. The measurements are based on spectroscopic techniques, simultaneously enabling the identification and quantification of various species, including alkanes and alkenes. Aromatics, however, cannot be directly measured. Therefore, an overview of emissions from an industrial site can be mapped relatively quickly compared to the DIAL method, considering the ability of this technique to detect numerous species simultaneously, at the cost of obtaining a more limited amount of information.
4. It is a costly but more affordable technique compared to using a DIAL system. A typical monitoring in an oil and gas plant may cost > €5000 per day, with a measurement duration of 8–10 days. However, the entire survey can take up to a month if the weather is not suitable for using SOF.
5. When applied to near-field measurements, this technique can serve as an effective tool for identifying major emission sources, although uncertainties in emitted flux measurements for single equipment are higher compared to using it on entire plants. SOF can potentially be used to provide better quantification of significant emitters. However, the resolution is not sufficient to allow for the identification of individual emission points.
6. Like DIAL, it allows the quantification of specific challenging sources. For example, it can determine emissions from flare combustion. If the flows and compositions sent there are known, it also allows measuring the efficiency of the flare itself and implementing the estimation of emission factors.

Regarding the current disadvantages of the SOF technique, the following can be stated:

1. The SOF technique uses the sun as a source of IR radiation. It can only be used during the day and only when sufficient sunlight is available for adequate measurement conditions. It is important to note that emissions from loading operations are typically higher during daytime working hours, with solar radiation further elevating certain VOC emissions, such as those from leaks in storage tanks.
2. The SOF technique offers a measurement of the average concentration of a compound across the entire atmospheric column between the sun and the spectrometer. It cannot, therefore, provide concentration details along the length of the column to allow the identification of individual sources.
3. Aromatic species cannot be directly measured with this technique. These compounds can be quantified using alternative methods to establish average concentration ratios relative to pollutants that are directly measured.
4. To obtain emitted mass fluxes, it is necessary to multiply the concentration data by the wind speed component at the height of the smoke column. This cannot be achieved de facto since the height of the smoke column is actually unknown. This error can be limited when measurements are taken at a distance of a few hundred meters to several km, due to more homogenous wind fields away from the high surface roughness present in an industrial site. However, in cases where the plant under study is surrounded by other industrial structures, this distant measurement strategy may not be possible. Near-source measurements can result in an overestimation of the emission flux. Since the emission column near the source may be in the downwind depression zone of the structure itself (i.e., building downwash), the wind field profile along the scan line will vary with distance and will be significantly different from that measured in an area with flat terrain.
5. Upstream emission data must be subtracted from those measured downstream of the investigated source. The SOF technique’s strategy involves driving the detection system around a plant while performing continuous measurements, both upstream and downstream. To reduce the uncertainty, several measurement circuits are necessary.
6. SOF measurements are performed for relatively short periods and only during daylight hours. These measurements can only provide a short-term “*snapshot*” of emissions with temporal variations. Data obtained through SOF can help identify possible significant emitters, but extrapolation to provide long-term estimates can lead to significant errors.
7. Only one company in the world commercially provides SOF measurements.

### 3.3. Tracer Correlation (TC)

**Figure 4:**
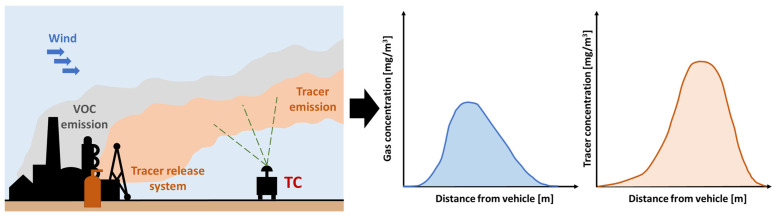
Operating scheme of the TC system.

The TC method is used to create a two-dimensional mapping of detected concentrations within an industrial site, enabling the quantification of gas emissions from major equipment such as tanks. Sometimes, it is also applied to entire specific sections of the industrial site if they are spatially isolated. This method is based on the controlled release of a known quantity of tracer gas, such as C_2_H_2_, N_2_O, or SF_6_. The concentration of the tracer gas is measured downwind, usually using a mobile device, while simultaneously measuring the concentration of the gas at the emission source [58,59]. Knowing the emission rate of the tracer gas at the source (kg/h) and measuring the concentration (mg/m^3^) in the downwind plume, both the tracer gas and the target emitted gas allow for the calculation of the emission flow. This technique assumes that the tracer follows the same advection and turbulent dispersion as the source gas and is primarily used to quantify emissions from already identified or suspected and isolated sources, source by source (Figure 4). For this reason, measurements must be conducted at a sufficient distance downwind of the source to ensure that the plumes of target and tracer gas are well-mixed [60].

For practical applications, in order to monitor general VOC emissions on an industrial scale, the TC system must meet several requirements in accordance with the EN 17628:2022 [31], as follows:

- The detection system must be securely installed on a mobile platform, with a sampling port facing the ambient air.
- It must include sensors capable of measuring individual VOC species or the sum of different VOC species to be estimated (e.g., alkanes, alkenes, alcohols, or aromatics) and, simultaneously, one or more tracer gas species.
- It must measure the tracer gas with a detection limit below 10 μg/m^3^, and for the source gas, above 20 μg/m^3^, while the vehicle is in motion.
- It must be able to conduct sampling for approximately 10 min, with a data detection frequency below 10 s.
- It must display real-time data on tracer gas and VOC concentrations, as well as the position of the mobile system on the map.
- It must include a tracer gas release device capable of maintaining a mass flow rate between 0.1 kg/h and 10 kg/h.

Furthermore, it is essential to obtain authorization for the release of the tracer gas near the sources under investigation. The choice of tracer gas is not critical in itself, provided that a high Release Precision Ratio (RPR) can be achieved, which indicates the strength of the downwind signal for a specific instrument and gas combination [61]. However, it is important to consider the costs and environmental impacts associated with different tracer gases. Moreover, all necessary safety measures must be adopted at the tracer gas release site, ensuring that no one is exposed to tracer gas concentrations above occupational hygiene limits. Examples of commonly used tracer gases are C_2_H_2_, N_2_O, and SF_6_. While the first is an unstable and flammable gas, the others have high and very high Global Warming Potential (GWP), respectively.

The mapping of the source gas concentrations must be performed using the TC sensor along all accessible roads of the site, monitoring the local wind direction to identify significant emission points and background levels. In detail, the plume should always be traversed at a distance that ensures both the adequate mixing of target and tracer gas and a clear distinction of the plume from the background concentration during the plume traverse [62].

The measured concentrations must be plotted on a two-dimensional map to identify any critical points and, if necessary, perform further concentration measurements using the TC sensor or additional techniques such as OGI or sniffers. The tracer gas must be released in the immediate vicinity of the identified sources, ensuring it disperses in the same way as the source gas and is located nearby.

Downwind gas concentration measurements should begin 10 min after the start of the tracer release to allow the tracer gas to mix adequately with the source gas and reach steady conditions. It is important to emphasize that the tracer gas should not be emitted in significant quantities from the site itself and must be chemically stable during advective transport.

The mass concentration ratio, along with the known tracer gas release flow (*Q_tracer_*), provides the emission flow of the source (*Q_VOC_*), expressed in kg/h. These flows are given for each individual section (*j*), integrating along distance from the emission point of the plume (*I*), as follows:

(2) $$Q_{VOC}^{j}=Q_{tracer}^{j}\cdot \frac{\int_{plumestart}^{plumeend}C_{VOC}^{j}dI}{\int_{plumestart}^{plumeend}C_{tracer}^{j}dI}$$

Finally, the success of the TC method depends on the correct co-location of the tracer and the emission source, as well as the need to accurately determine the tracer emission flow. For the proper placement of the tracer gas release system, it is necessary to preliminarily analyze the prevailing wind directions, assess accessibility and the presence of roads near the emission source at a distance sufficient to ensure the well-mixing of the plume, and evaluate the complexity of the terrain surrounding the emission to be quantified [63,64]. Moreover, it is crucial to ensure the stability of the tracer gas flow rate emitted by the release system. To this end, Scheutz et al. [62] recommends continuously monitoring both the emitted flow rate and the loss in weight of the cylinders employed.

Following the critical analysis of the standards and the available literature, the advantages and disadvantages of the TC technique can be highlighted. In particular, the advantages include the following:

1. It is a well-established technique, particularly when the source is isolated and the aim is to measure or verify the mass flow rate emitted.
2. With a known release from a particular source, there are no significant issues attributable to the upwind contributions with respect the emission source.
3. Various tracers are available, sometimes identifiable in the emission itself given the composition of the plume.
4. The technique is relatively low-cost, defined mainly by the placement of the controlled release system for the tracer rather than the instruments used to detect ground concentrations (Photoionization Detector, i.e., PID, or Flame Ionization Detector, i.e., FID).

On the other hand, the disadvantages of the TC techniques include the following:

1. The potential source must be known for the placement of the controlled release system for the tracer.
2. Significant calculation errors in the evaluation of the emission flow are possible, particularly due to the definition of the wind speed profile, especially near the source, where the flow is very complex.
3. The tracer must be compatible with worker health and the site’s production. For accurate measurement, normal plant operation must be ensured.
4. The technique is not suitable for chimneys or high-altitude sources, given the difficulty of evaluating ground concentrations of the tracer near the source.
5. It is impossible to demonstrate the hypothesis underlying the method, i.e., the tracer follows the same advection and turbulent dispersion as the source gas.

### 3.4. Optical Gas Imaging (OGI)

The OGI method involves the usage of an IR camera, equipped with an optical filter sensitive to the IR absorption bandwidth for specific VOCs [64,65,66]. This camera allows the observation of VOC plumes, which appear as clouds on the display. The primary aim of OGI is to detect and locate emission sources, providing details about their positions, which can later be quantified using other methods such as DIAL and SOF. However, OGI does not quantitatively determine the emission flows from leaks or emissions. In recent times, despite not being formally mentioned in the EN 17628:2022 standard [31], the Quantitative Optical Gas Imaging (QOGI) method has been introduced. This system enables the quantification of emissions flows by coupling an OGI camera with dedicated software for analyzing the obtained thermographic images.

OGI applications include the following: identifying emissions from a safe distance (using various lenses), detecting unwanted emissions from sealed devices, locating leaks on components and tank joints, monitoring emissions during loading operations, and conducting qualitative inspections post-maintenance.

Before using OGI, it is essential to evaluate the production process to determine the lines or equipment to be inspected, the composition of the fluids contained, and the IR spectra to consider for detecting emissions.

OGI application procedures include the following:

- Recording emissions from variable sources for at least 20 s or as long as necessary to detect variability.
- Possibly acquire a video to capture the entire plume and surrounding context.
- Creating a visible image (non-IR) of the emission source.

No specific calibration of the camera is required for OGI, but a functional test is necessary to validate its performance. This test involves verifying the camera’s ability to detect a specific substance from a certain distance under specific weather conditions. Since atmospheric conditions and the composition of VOCs affect OGI sensitivity, the test should be performed under real conditions and may need to be repeated if conditions change during the investigation.

However, OGI has several limitations, as follows:

- It requires high VOC concentrations and an appropriate background for plume visualization; it may not effectively detect very diluted emissions or sources subject to rapid dispersion.
- It cannot detect emissions from large equipment or plant sections due to plume dilution.
- Emission quantification is theoretically impossible with OGI, which only provides a qualitative assessment of potential emission sources.

Several OGI cameras are available commercially, with price variations based on the range of detectable compounds and camera resolution. Currently, the market leader in such equipment is FLIR [67], particularly the Gx320 camera version.

OGI technology can detect a wide range of chemical species, including light aliphatic hydrocarbons, olefins, and aromatics, which account for the majority of VOC emissions. The instrument is not intended to be selective for a single compound but to detect, to some extent, all chemical components present in leaks in refineries or petrochemical plants, methane included.

Laboratory tests conducted in wind tunnels [68,69,70] have shown that OGI sensitivity depends primarily on the molecule type and wind speed. These tests revealed that the detection limits for light aromatic compounds are generally higher compared to those for light aliphatic and olefinic hydrocarbons. Detection limits increase with wind speed. The best currently available technology, the FLIR Gx320 camera, has a detection limit for methane of 0.6 g/h, sufficiently low for detecting typical real-world emission flows. CONCAWE conducted a controlled leak test in the spring of 2003 at the LDAR training and study facility, which allows the generation of leaks of various sizes and from different types of equipment. During field campaigns, the detection limit of the FLIR Gx320 camera was tested. The aim was to determine which flow values (g/h) were visible using the IR camera from 2 m away, in normal or high-sensitivity mode [71].

Based on available documents, the advantages and disadvantages of the OGI technique can be highlighted. In particular, the following advantages can be noted:

1. Experience shows that a team of two people using an OGI camera can typically inspect about 2000 equipment components per day. This performance is primarily influenced by the time needed to tag components identified as leaks for repair, as the camera operator must relay the location to the assistant. Conventional sniffing techniques, i.e., using PID/FID, are about four times slower (i.e., approximately 500 equipment components can be checked per day).
2. All equipment components can be checked. This allows for the detection of large leaks in non-accessible positions, which would remain undetected in conventional sniffing monitoring.
3. Current OGI cameras are the size and weight of household camcorders. This allows these instruments to be carried into process areas and tank roofs, which is not possible with more complex systems.
4. Two or three days of training are needed to enable the use. Unlike sniffing, the camera does not necessitate instrument calibration, which consequently lowers the required skill level for the operation.

On the other hand, the disadvantages of the OGI technique are as follows:

1. The OGI technique is less effective than conventional sniffing methods in rain or fog. It also loses effectiveness in the presence of limited temperature differences with the surrounding environment.
2. The price of commercially available camera systems varies from €40,000 to €100,000. In contrast, VOC detectors used in conventional sniffing surveys range from €5000 to €25,000, depending on their complexity. However, multiple detectors are often required to conduct a comprehensive site survey within a short timeframe.
3. OGI cameras are generally not fully ATEX-rated, and work permits require the use of an explosion meter to check inspection areas. Conversely, detectors used in conventional sniffing surveys are rated for use in hazardous areas.

#### Quantitative Optical Gas Imaging (QOGI)

An advance on using OGI for detection alone is the development of a new methodology for obtaining an emission rate from an OGI thermographic image [67]. The Quantitative Optical Gas Imaging (QOGI) system uses a conventional OGI camera, coupled with a proprietary software capable of processing the thermography and providing a quantification of the emitted mass flow rate. Before QOGI, OGI techniques were effective visual tools but had limited application for LDAR compliance, as they were only qualitative. Consequently, the American Petroleum Institute (API) developed OGI loss/no-loss [72] factors to enable the quantification of VOC emission rates from leaks detected by an OGI camera. However, the application of the QOGI technique is not strictly reserved for the oil and gas sector; in fact, this technique is particularly effective for identifying and quantifying fugitive emissions from landfill biogas collecting system [73] and biogas production plants [74].

**Figure 5:**
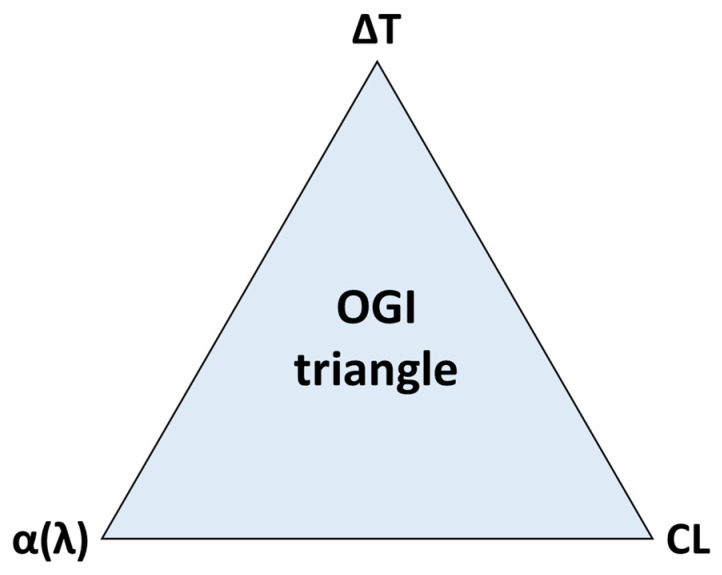
Operating parameters of a QOGI system.

Two parameters influence the QOGI system’s performance in quantifying emissions [75] (Figure 5): the temperature difference between the released gas and the background (ΔT) and the total number of molecules along a line of sight through the gas column to the background, referred to as concentration path length (CL). This integrated path concentration length is measured using the brightness of each pixel in the image. The system has detection/quantification limits represented by a minimum CL value, and this minimum value is expected to decrease as ΔT increases. Another parameter, considered fixed in the first approximation, is the camera’s sensitivity to the absorbed wavelength. This is set using a specific wavelength filter on the camera matched to the target gas. Since many hydrocarbons have similar absorption spectra, response factors can be used to account for different gas compositions.

The quantitative module uses proprietary algorithms to analyze IR images of a leak, pixel by pixel, to automatically calculate mass emission rates, in terms of g/h. Each pixel corresponds to a column of hydrocarbon vapor situated between the camera and the background. The contrast intensity of each pixel is determined by the temperature difference (ΔT) between the background and the vapor column. At a specific ΔT, the intensity of the contrast is directly proportional to the number of hydrocarbon molecules present in the vapor column.

The QOGI system requires the user to provide the ambient temperature and the distance from the OGI camera to the leak. In cases where ΔT is insufficient to apply the method, the background can be enhanced to generate the necessary temperature differential. This enhancement can typically be achieved by applying a heated or cooled surface behind the leak.

**Figure 6:**
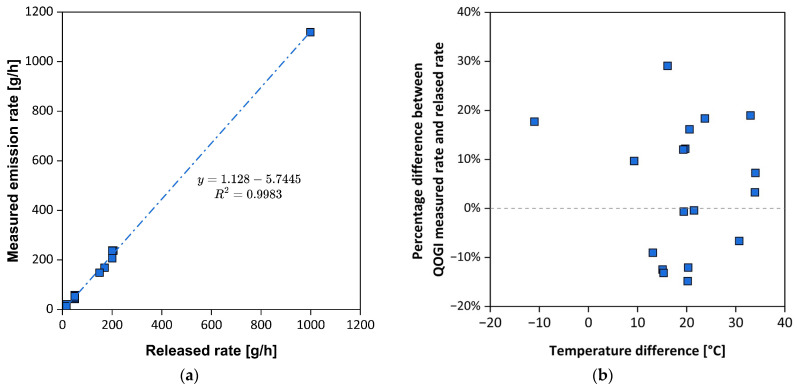
(**a**) Comparison between emission rate measured (QOGI) and released. (**b**) Percentage difference between measured (QOGI) and released flow as function of the temperature difference between emission and background.

In 2015, CONCAWE [71] conducted studies to verify the validity of the OGI system using an experimental setup capable of simulating fugitive emissions from a leak. This study aimed to simulate the controlled release of propane, methane, propylene, and a mixture of these VOCs at a known flow rate and then compare it with the QOGI results. In the preliminary study, various parameters were varied, including the type of component from which the leak occurred, the background material, the distance from the source, and the ΔT between the emission and the background. The results indicated that the key parameter for visualizing and quantifying the plume is ΔT, which must be greater than 5 °C for effective resolution. The ideal distance from measurement was found to be around 3 m. Under these “*ideal*” conditions, the validation study was conducted. Figure 6a shows the comparison between the emission rate quantified with the QOGI system and the controlled mass flux released, while Figure 6b shows the percentage difference between measured and released flux as a function of ΔT between emission and background.

**Figure 7:**
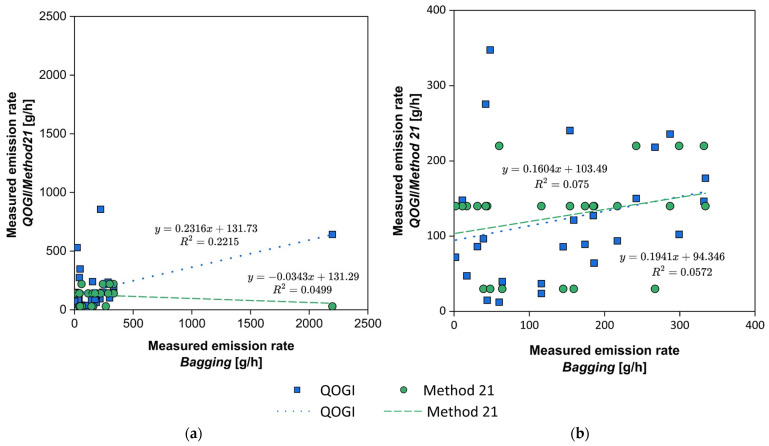
Comparison between different techniques and methods for estimating fugitive emissions: (**a**) considering all the available data and (**b**) considering only measured emission rates lower than 400 g/h.

In 2020, CONCAWE conducted a field study to validate the QOGI technology [76], by comparing its performance to traditional methods, such as *Bagging* and the Screening Range Approach outlined in EPA’s Method 21, which relies on SOCMI Screening Range Emission Factors [77]. Following an LDAR campaign aimed at the identification of the primary sources of fugitive emissions at the facility, emission quantification was carried out using these techniques. The findings of the study are presented in Figure 7.

Figure 7a shows that the QOGI technique provides an estimate with reasonable adherence to one measured using the Bagging technique, whereas the Screening Range Approach outlined in EPA’s Method 21 shows a grossly independent trend as the measured emission rate increases. This result can be due to the fact that a fixed concentration value (*pegged value*), equal to the full scale of the FID used during the LDAR campaign, was used when the leak exceeded the instrument’s maximum detection concentration, as indicated by the EPA protocol [77]. In this way, the value obtained with the Screening Range Approach of Method 21 depends exclusively on the type of source analyzed, regardless of the actual concentration emitted. Focusing on Figure 7b, where measured emission rates exceeding 400 g/h have been excluded, comparable regression lines between QOGI and Method 21 can be observed. This suggests that for lower emission rates, both methods appear consistent with the Bagging technique. Additionally, the data distribution in the graph reveals that the use of QOGI enables greater stratification of the measured data compared to the Screening Range Approach of Method 21. Specifically, the QOGI method provides more representative data, particularly when measuring concentrations exceeding the upper *pegged value* of Method 21.

Currently, for the effective use of the QOGI technique, further laboratory-scale studies appear to be necessary to effectively test additional VOCs beyond the already tested propane and methane [78] and to further investigate the multiple variables involved (e.g., type of background, ΔT between emission and background, emission rate, or distance from the emission). It appears that there is an absence of wide range validation studies conducted by independent bodies.

### 3.5. Reverse Dispersion Modelling (RDM)

Atmospheric dispersion modelling tools are widely used and validated for predicting the spread of pollutants in the air. Specifically, inverse modelling (RDM) leverages these tools by comparing observed concentrations in the fallout of an emission source with those predicted by the model [79]. Initially, a direct dispersion simulation is performed, assuming a hypothetical emission rate. Subsequently, the actual emission is estimated by linearly adjusting the emission mass flow so that the predicted concentrations match the measured ones. This approach, standardized for measuring dust mass flows in EN 15445:2008 [80], can be extended to monitoring various pollutants, provided atmospheric concentrations can be measured.

To monitor hydrocarbon mass flow, the analyzer used in RDM must be suitable for the purpose. For VOCs, for instance, a portable FID/PID analyzer may suffice, but other point measurement techniques can be used depending on the pollutant. No specific calibration is required for RDM, except for the concentration analysis instrument. Regarding wind field information, it is essential to have a meteorological station near the study area, equipped with wind sensors and the capability to measure the parameters needed to calculate atmospheric turbulence. Expeditiously, to perform RDM, a portable FID analyzer can be used alongside a 3D ultrasonic anemometer placed at a height of 10 m.

The choice of the monitoring area depends on various factors, such as wind conditions and accessibility around the investigated sources. The RDM technique involves an initial screening of concentration in the emission area to identify emission points and source characteristics. Subsequently, measurements are taken around the emission sources, with at least five points within the main plume.

EN 17628:2022 standard [31] prescribes that, if possible, measurement should be conducted with winds exceeding 2 m/s (measured at 10 m above ground level), including measurement points both upwind and downwind of the emission source. If multiple sources are present in the area, the measurement path must include points around each one. Sampling time varies from 30 to 60 min, depending on the model used.

RDM uses two phases to obtain the emission flow: a direct dispersion simulation phase and an inverse modelling phase to estimate the actual emission. This involves calculating concentrations at each monitoring point and using linear or multilinear regression, depending on the number of sources.

A crucial step to ensure high accuracy in the results of an inverse modelling study is the appropriate filtering of data. For instance, in low wind conditions, there is typically a decrease in the accuracy of emission estimates. These aspects were experimentally investigated by Flesch et al. [81], who analyzed in depth the effect of data filtering based on various parameters, such as friction velocity, temperature gradient, and atmospheric stability, during both daytime and nighttime hours. Specifically, it was observed that filtering data solely based on friction velocity is sufficient for daytime conditions. However, to achieve high accuracy during nighttime, it is also necessary to filter the data based on the temperature gradient.

Particular challenges in the application of the RDM technique are represented by areal emission sources, such as wastewater treatment tanks, or sources located in non-ideal terrains (e.g., undulating ground or presence of buildings). To address these complexities, several experimental studies have been conducted to ensure the accurate application of inverse modelling in such complicated scenarios. Specifically, some recommendations are as follows:

- Avoid measuring concentration data too close to the emission source, considering a distance of at least 10 times the height of the emission source [82].
- The distance of the concentration detector from the source should be such that it captures a noticeable variation from the background concentration [82].
- Periods of high atmospheric stability may lead to a loss of accuracy in the emission estimates and should be therefore excluded [82].
- Simply increasing the number of detectors and concentration measurements is not sufficient to guarantee an improvement in emission estimate accuracy. A key factor is the proper placement of instruments based on a preliminary study of the meteorological conditions [83,84].

The location of the modeled source must be known and possibly supported by methods such as OGI. To accurately interpret the data, it is necessary to use statistical algorithms and ensemble learning analysis, i.e., using multiple models or algorithms to achieve better predictive performance than those obtained by individual models applied separately.

Following a critical analysis of the regulations and the available literature, the advantages and disadvantages of the RDM technique can be summarized. In particular, the following advantages can be reported:

1. It is a well-established technique, already used and internationally regulated (EN 15445) for dust dispersion evaluation. Additionally, atmospheric dispersion models are widely used in evaluating the ground-level impacts of industrial emissions.
2. It has relatively low costs compared to other techniques mentioned in this document, mainly attributable to ground concentration field detection using a traditional sniffing method (PID/FID).

On the other hand, the disadvantages of the RDM technique are as follows:

3. The modeling is heavily influenced by the provided meteorological data, particularly wind direction and speed, and ground-level concentration measurements.
4. It is not possible to distinguish contributions from potential upwind sources in the observed area.
5. Correct model implementation requires source localization, using other detection and identification techniques.
6. In the presence of multiple sources or even multi-company sites, obtaining a single flow of data requires simultaneous data for each source. This is characterized by significant practical difficulties, both in locating each source and in measuring the various concentration values needed.
7. It is not suitable for evaluating high sources, due to difficulty in measuring ground concentrations near the sources.

## 4. Summary and Comparison of the Described Techniques

Following a comprehensive analysis of the techniques outlined in the EN 17628:2022 standard [31], highlighting their strength and limitations, a comparative analysis is employed to provide a clear guidance on the selection of the most suitable technique for the specific context under consideration, e.g., estimation of diffuse emissions from a single equipment or an entire plant section. Table 1 summarizes the key parameters for each technique, including the measurement scale and time, type of sources and target compounds, role, output, detection limits, indicative costs and associated uncertainty.

Following the presentation of the key characteristics of the monitoring techniques described in the EN 17628:2022 standard and the innovative QOGI technique, summarized in Table 1, it becomes evident that each method is suitable to specific operational contexts depending on the measurement scale, emission sources, and monitoring objectives. Techniques such as DIAL and SOF are particularly effective for monitoring plant sections and entire industrial facilities, offering the ability to detect and quantify emissions over medium to long distances, making such techniques suitable candidates for estimating diffuse emissions. The use of DIAL enables the creation of geolocated concentration maps with measurement accuracy ranging between 5% and 25%, providing both quantification and localization of emission points. Conversely, SOF allows for monitoring on a larger scale (up to 10 km) but with reduced accuracy (21–37%). The RDM technique uses theoretical atmospheric dispersion simulations to quantify diffuse emissions from major plant equipment (e.g., wastewater treatment plants, tanks, or entire plant sections), leveraging well-established measurement tools (PID/FID). A critical aspect for the effective application of this method is the selection of the most appropriate dispersion model based on the configuration of the emission source. The TC method is valuable for monitoring isolated point or area sources by employing gas tracers; however, its successful implementation requires careful management and proper positioning of the tracer release system, as well as a precise localization of the potential fugitive emission. Finally, the OGI technique, instead, is most suitable for the inspection of specific components, such as valves and pumps, due to its ability to allow for the visualization of leaks in real time. Although it does not enable the quantification of emission fluxes, its rapid detection facilitates prompt repair of the leakage. This limitation of the OGI technique is completely overcome by the implementation of the quantitative algorithms (QOGI), making it ideal not only for inspections but also for estimating fugitive emissions from individual equipment components. Thus, the choice of the most suitable technique depends on multiple factors, making a case-by-case evaluation essential to optimize monitoring operations. A key aspect to consider is also the economic feasibility of these techniques; it is important to highlight that the costs estimates of a monitoring campaign presented in Table 1 are purely indicative. In fact, the costs associated with these techniques depend on various factors, including the monitoring objectives, the area to be monitored, the duration of measurement campaigns, and meteorological conditions, as well as the limited commercial availability. Furthermore, it should be noted that all the techniques are of discrete nature, meaning that the data obtained cannot be extrapolated beyond the monitoring period, thus increasing the costs for a comprehensive characterization of the emission scenario. A potential solution could involve the use of machine learning algorithms to make the individual monitoring efforts more robust under specific production and environmental conditions [100,101,102,103].

## 5. Review of Case Studies and Application of Techniques

### 5.1. Chronology of Comparative Studies Conducted

For a better understanding of the methodologies proposed within the EN 17628:2022 standard [31], several case studies dedicated to comparing techniques for quantifying fugitive and diffuse VOC emissions are presented in the following paragraph. Specifically, starting from the data reported in these studies, a critical analysis of the results obtained was conducted to evaluate the effectiveness and limitations of the techniques proposed in the standard. Table 2 lists the various considered case studies, specifying each site of analysis, the coordinating institution, and the companies and techniques involved in the study.

One initial consideration regards the publication years of the studies considered: although these are innovative technologies, the most recent literature studies date back to 2017, while current research is primarily focused on the optimization and development of the QOGI technique [75,77,98].

### 5.2. Comparison of Techniques with Controlled Release

To better understand the functioning of the techniques described within the EN 17628:2022 standard [31], data collected during field measurement campaigns for the validation of techniques on the field, organized by Working Group 38 (WG38) [105], the working group that drafted the standard in question, and Veolia Environment Research & Innovation (VERI) [107], were analyzed. Specifically, the data collected by the various participants of WG38 were reprocessed to evaluate the adherence of the measured data in a controlled release situation. Subsequently, data obtained from Babilotte et al. (2010) [107] were integrated to gain a logistic–economic interpretation regarding the use of optical techniques.

The field validation program conducted by WG38 was carried out at a decommissioned refinery in southern France in June 2016: a Controlled Release Facility (CRF) system, developed by NPL and managed by the Institut National de l’Environnement Industriel et des Risques (INERIS) as the project manager and meteorological sensor installation manager, was used on that occasion. The gas released was mainly propane (about 93%) with a small percentage of propene, i-butane, and n-butane.

The participants in the measurement campaign were the following:

- Bureau Veritas, as the operator of the OGI technique.
- NPL, as the operator of the DIAL technique.
- FluxSense and Chalmers University, as the operator of the SOF and TC techniques.
- Total, as the operator of the RDM technique.

**Figure 8:**
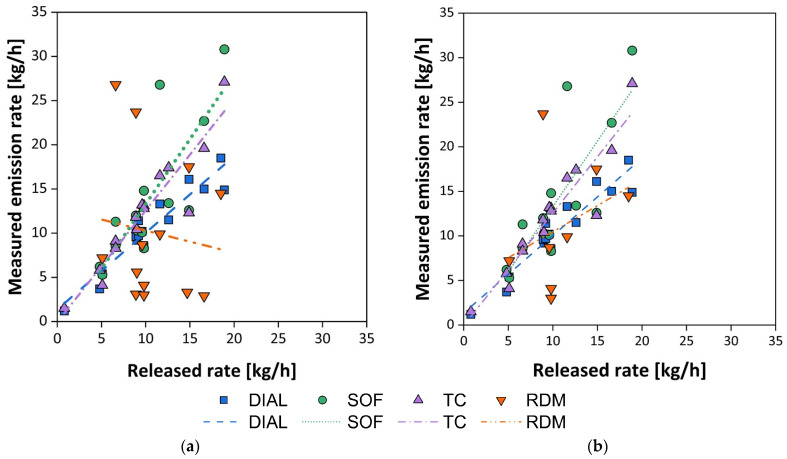
Comparison between measured and released emission rates by the CRF system during the various campaigns conducted: (**a**) considering all the available data and (**b**) considering only data with speed greater than 2 m/s for RDM technique.

Figure 8a shows the data obtained by the various actors involved, highlighting the trend of the measured emission rate as a function of the released emission rate. Trend lines for each adopted technique are also shown in the graph, while Table 3 provides the parameters of the regression lines performed. For the TC method, the data obtained by comparing the released and measured tracer gas flow, i.e., N_2_O, are reported.

As can be easily observed, the DIAL, SOF, and TC techniques show reasonable adherence between measured and released data (R^2^ > 0.65), while the RDM technique presents very poor data adherence: a possible explanation for this behavior can be attributed to the dispersion model adopted by Total, i.e., a combination of a CFD model and a Lagrangian dispersion model (SLAM, i.e., Safety Lagrangian Dispersion Model). Indeed, excluding the data measured under wind speed conditions below 2 m/s, as also suggested by Flesch et al. [81], it is observed (Figure 8b) that the RDM technique also shows a consistent trend in measuring the emission rate: in statistical terms, although the determination coefficient (R^2^) increases from 0.01 to 0.11, an increasing trend consistent with the expected results is observed (Table 3).

In general, excluding the RDM technique, a bias towards a positive estimation of the flow compared to the released flows can be observed. The controlled release was characterized by flows varying from 0.18 to 0.8 kg/h, which are typical flows of the smaller emission sources and are generally detectable with standard methods (i.e., sniffing) once the location is known. Despite this, the RDM technique was unable to measure emissions below 5 kg/h, while the OGI technique provided valid indications for a qualitative evaluation for each emission test.

Examining the collected data, a particular sensitivity of the adopted techniques to meteorological conditions, especially in terms of wind speed, is evident: in calm wind situations (U < 0.5 m/s), no method was able to obtain a valid measurement. Indeed, under calm wind conditions, the emitted gas tends to stagnate near the emission source, resulting in a non-uniform distribution of concentration [109,110]. Furthermore, traditional concentration detectors (such as those involved in the application of TC and RDM) require minimum airflow to aspirate and transport the gas sample to the sensor [111].

Moreover, analyzing the setup of the validation campaign [107], it is observed that each operator placed their meteorological sensor in different positions: this aspect is reflected in different wind speed values for each technique considered, despite the simultaneity of the measurements. Therefore, as observed from the previous analysis, the data obtained with each technique are strongly influenced by the choice of the reference meteorological sensor.

**Figure 9:**
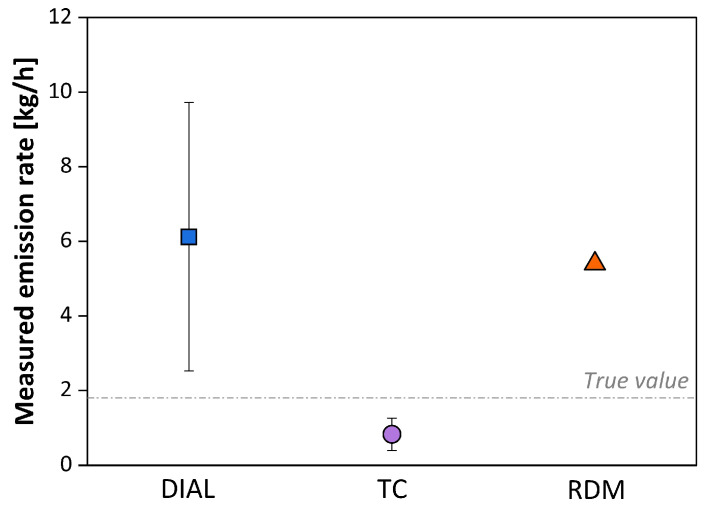
Comparison of methane emission rate measured with different techniques (markers) and the controlled released rate (dash-dotted line) [107].

Substantially similar results and considerations were also found in the study conducted by Babilotte et al. (2010) [107] in October 2007 at a municipal solid waste landfill in southern France, where a controlled release of methane was carried out (Figure 9).

The obtained results clearly show that the DIAL technique, the most expensive and complex, provides the best estimate of the emission rate. This is consistent with a theoretical principle of the technique’s operation, having a defined target compound, i.e., methane, to detect and quantify. The results obtained with other methods are less satisfactory; in particular, in the case of inverse atmospheric modeling, RDM, situations of calm wind were recorded as in the WG38 case study [105].

An interesting aspect highlighted in the VERI publication [107] concerns the economic aspects, timings, and logistics related to each adopted method, detailed in Table 4.

### 5.3. Comparison of Techniques in a Field Situation

Following the analysis of data collected from a field validation of the techniques proposed by the EN 17628:2022 standard [31], a detailed investigation was carried out on the results obtained using optical methodologies to evaluate emissions from unknown real sources. In this section, data from various campaigns [57] conducted between August 2000 and July 2005 at the AB Nymäs Petroleum refinery in Nynäshamn, Sweden, and at the Esso Netherlands B.V. refinery in Rotterdam, Netherlands, in March 2017 [105], are collected. As the true value of the emission flow was not known, it was only possible to make a qualitative comparison between the techniques.

In the Swedish study, before evaluating the overall VOC emissions of the plant, preliminary measurements were taken using different techniques near a precise source, but without knowing the characteristics of the flow (in terms of rate and composition). Specifically, the source considered was the vent of a vapor collection duct from ten heated tanks containing bitumen.

**Figure 10:**
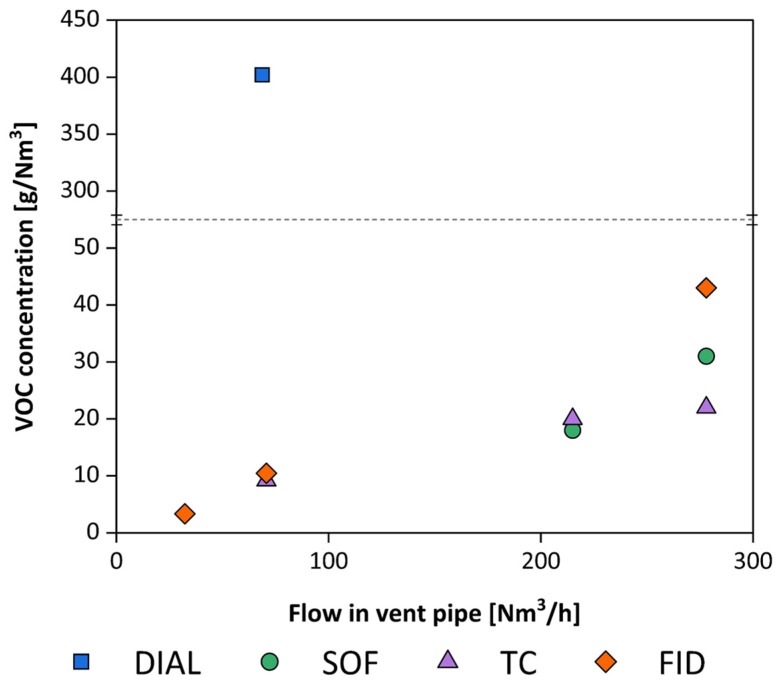
Comparison of results obtained with different techniques in campaigns conducted in Sweden (note the discontinuous values on the y-axis).

Figure 10 shows the data obtained from various campaigns, as VOC concentration was measured as a function of the airflow rate of the duct.

An immediate observation is the aberrant data measured with the DIAL: project managers assumed that the instrument was not properly set up for quantifying gases emitted from the source under examination. In fact, excluding the data obtained with the DIAL, the measurements taken with the other techniques yield, all in all, comparable results, despite being obtained in different situations. The data collected in the figure were obtained under different meteorological conditions.

However, regarding the quantification of VOC emissions from an entire refinery, the campaign conducted in the Netherlands in 2017 [105] showed significant variability in the outcomes. The collected data did not permit conclusive assessments regarding the effectiveness and reliability of the techniques employed in quantifying emissions across an entire industrial site; the measurements were conducted inaccurately for proper reproduction. For example, some estimates were provided by summing measured contributions and calculated contributions (e.g., API Tanks) under different meteorological conditions.

It should be noted that the results shown as annual VOC emission estimates from refineries in the 2008 CONCAWE report [57] were extrapolated from a DIAL campaign lasting two hours, reinforcing the consideration that extrapolating short-term data to annual evaluations can lead to significant overestimates of flows.

The use of the techniques described in the EN 17628:2022 standard is not exclusively limited to large industrial facilities, such as refineries or petrochemical complexes. In fact, these techniques can also be applied, for instance, to estimate methane emissions from livestock farming [106] or from a biogas production plant [108]. The use of TC and RDM techniques has actually been widespread for a long time, even prior to the introduction of EN 17628:2022 standard.

Referring to the study conducted by Bai et al. [106], in February 2013, the TC and RDM techniques were employed to estimate methane emissions from a beef cattle farm in Australia. Specifically, the use of tracer gas in such context is well-documented in the literature [112,113], yet challenges persist in the proper placement of the tracer gas release system (N_2_O in the mentioned case study), due to the continuous movement of the cattle within the fenced enclosure. However, by applying the RDM technique, it was possible to consider the entire fenced enclosure as a single emission source. A comparison of the results obtained from the two techniques employed suggests that RDM offers a valid alternative to TC, particularly in reducing the logistical challenges associated with the implementation of a tracer gas release system.

Additionally, the study conducted by Hrad et al. [108] between October 2016 and May 2017 at two different biogas production plants in Austria, provides clear guidance on the selection of the most appropriate technique based on the monitoring objective. The research findings suggested that methods such as DIAL, TC, and RDM, referred to in the study as “*off-site*” methods, are more effective for quantifying diffuse emissions from an entire facility compared to on-site methods (including OGI and FID detectors), particularly when the localization of multiple sources is unknown and operational conditions are variable. Specifically, DIAL and TC are more suitable for short-term campaigns, while RDM is better suited for long-term monitoring [114].

### 5.4. Comparison of Techniques on Tank Measurements

Despite the evident challenges associated with the use of optical techniques for accurately estimating VOC emissions from an entire plant, given the numerous variables of interest and the inherent complexity of the individual methods, several measurement campaigns have been conducted to quantify emissions from tanks.

In this section, the main results related to the comparison of VOC emission quantification from storage tanks using DIAL and TC techniques [57,104] versus estimates with API methods [115,116] are reported.

In January 1995, a study was conducted using the DIAL technique on a floating roof tank at a refinery that contained crude oil. The DIAL measurement, lasting 4 days, allowed the return of 73 valid scans to be compared with the diffuse emissions estimate obtained using API algorithms, after verifying the integrity status of the tank, and so the comparability with API estimates. A 4-day measurement period was chosen to simulate a complete filling and emptying of the tank.

This study clearly demonstrated that, as the number of scans performed with the DIAL increases, the emission rate quantification becomes increasingly comparable to the API estimate. Indeed, measuring in different states of the tank allows “*smoothing*” of the instantaneous data. The obtained results are very significant, as achieving a representative estimate with the DIAL technique requires a high number of scans, causing an increase in the costs and time required for the measurement.

Another fundamental variable for evaluating the representativeness of flow estimates using API algorithms is the state of the tank. To this end, a study [57] was conducted in June 2007 at the Shell refinery in Gothenburg, Sweden, with the aim of reducing diffuse emission from tanks. This study involved the use of the OGI technique to evaluate the state of the tanks and identify any leaks and discontinuities, in addition to the TC technique to quantify emissions.

Once the main leaks were identified, the tracer gas release system (i.e., N_2_O) was positioned near the emission point. The data obtained with the TC technique were then extrapolated to obtain the annual VOC emissions estimate to be compared with the annual estimates provided by API algorithms [115,116]. Table 5 shows the characteristics of the considered tanks and the results of the API–TC comparison.

The results show that the VOC emission estimates using API algorithms and the measurements taken with TC may not always be comparable. Indeed, closely observing the screening carried out with OGI cameras highlights that the API estimation methodology is robust as long as the tanks are in good condition. This outcome aligns with the specifications outlined in AP42, Section 7.1, which clearly emphasize that “*The methodologies are intended for storage tanks that are properly maintained and in normal working condition*” [117]. As already mentioned, OGI may be a very useful tool in order to maintain industrial equipment prior to conducting a proper monitoring campaign.

## 6. Conclusions

The recent publication of EN 17628:2022 represents an important step forward in the standardization of techniques for the localization, identification and, most importantly, quantification of diffuse VOC emissions from industrial sources. Indeed, the proposed techniques, especially those based on optical instruments, show high potential for the detection and quantification of emissions that are typically difficult to detect by traditional methods. However, these techniques, although well-established, are particularly complex both from a technical–scientific and logistical–economic point of view. In fact, applicative case studies have shown a strong dependence on environmental variables, such as wind profiles, requiring special attention in the implementation of these data to ensure reliable results.

In addition, the analyzed data, resulting from an extensive literature search, suggest that each technique proposed in the regulations has specific fields of application, each with its own advantages and limitations that affect its effectiveness. Methods such as DIAL and SOF appear particularly suitable for mapping and quantifying large-scale diffuse emissions, although cost and operational complexities could pose significant obstacles to optimal application of the aforementioned techniques. In contrast, more affordable techniques such as OGI and QOGI offer an optimized approach for the localization and quantification of the emissive sources, but further studies are necessary for reliable estimation of the emitted fluxes. Finally, the TC and RDM methods appear useful in specific contexts, particularly the former, if the emissive source is well localized, and the latter is more effective under simple meteorological conditions and with isolated sources.

In conclusion, the efficacy of a diffuse VOC emission monitoring program, in accordance with EN 17628:2022, depends on a meticulous selection of the most appropriate technique for the particular circumstances under consideration. The selection of the proper technique must be based on a robust definition of the objectives, a clear understanding of the characteristics of the emission sources, and logistical feasibility. Furthermore, the analysis of the advantages and limitations of the techniques reported in the standard reveals the necessity of integrated approaches that combine multiple techniques to achieve a representative characterization of diffuse VOC emissions from an industrial site. This review aims to help environmental engineers and researchers in selecting appropriate methodologies for gas monitoring, thereby contributing to more effective and reliable gas monitoring and, therefore, emission control strategies.

## Figures and Tables

**Table 1:** Comparison of the key parameters of the techniques described in the standard.

Parameter	DIAL	SOF	TC	RDM	OGI	QOGI
Measurement scale	100 m–1 km (max resolution = 3 m)	10 m–10 km (max resolution = 10 m)	10 m–2 km (max resolution = 10 m)	Theoretically no limitation (in accordance with the model used)	<1 m	<1 m
Type of sources	Main plant equipments, plant section	Main plant equipments, plant section, entire industrial site	Main plant equipments	Main equipments, plant section	Components (pumps, valves, flanges, seals, …)	Components (pumps, valves, flanges, seals, …)
Role	Detection, localization, quantification	Detection, localization, quantification	Detection, quantification	Detection, quantification	Detection, localization	Detection, localization, quantification
Output	2D concentration map in ppm, emissive mass flux in kg/h	Geolocated concentration columns in mg/m^2^, emissive mass flux in kg/h	Geolocated concentrations in μg/m^3^, emissive mass flux in kg/h	Concentration in mg/m^3^, emissive mass flux in kg/h	Plume visualization	Concentration in ppm∙m, Emissive mass flux in g/h
Measured compounds	Benzene, toluene, hydrocarbons C3+ (max two compounds at a time)	Alkanes (C2–C20), alcohols (C1–C8), alkenes (C2–C4), amines, aldehydes, dienes	Sensor selected based on the tracer (e.g., C_2_H_2_ or N_2_O)	No limitation (traditional PID/FID detection tools)	No complete list of detectable compounds is currently available (e.g., methane, benzene < 50 ppm∙m; ethylene, phenol < 150 ppm∙m)	No complete list of detectable compounds is currently available (e.g., methane, benzene < 50 ppm∙m; ethylene, phenol < 150 ppm∙m)
Measurement time	~15 min per single scan (at least four scans required for a single measurement)	1–2 s per single column (about 20 m base)	1–5 min for point sources, 10 ÷ 20 min for areal sources	30–60 min	About 1000 components per day per operator	About 1000 components per day per operator
Detection limits	30 ppm in IR, 10 ppb in UV	1–5 mg/m^2^ for concentrations, 1 kg/h for mass flux	10 μg/m^3^ for concentrations, 0.1 kg/h for emissive flux	Function of the configuration of the emission source	0.8 g/h for methane	0.8 g/h for methane, 3.8 g/h for toluene, 0.7 g/h for ethanol
Measurement uncertainty	5–25%	21–37%	20–40%	Largely dependent on meteorological conditions	No quantification	Not yet determined
Indicative costs of monitoring	100,000–200,000 €	50,000–150,000 €	20,000–70,000 €	30,000–100,000 €	10,000–50,000 €	25,000–60,000 €
References	[42,43,85,86,87]	[56,88,89,90,91]	[60,92,93,94,95]	[82,84,96,97,98]	[68,69,70,71,99]	[67,72,73,74,75]

**Table 2:** Available comparative studies of the described techniques present in the literature.

Date	Location	Organizing Body	Techniques Involved	Reference
January 1995	Not specified	CONCAWE	API algorithms, DIAL	[104]
Autumn 2000	Sweden	CONCAWE	API algorithms, DIAL	[57]
June/July 2005	Sweden	CONCAWE	SOF, TC, FID	[57]
June 2007	Sweden	CONCAWE	API algorithms, OGI, TC	[57]
October 2007	France	Veolia Environnement Reasearch & Innovation (VERI)	TC, DIAL, RDM	[105]
February 2013	Australia	Australian Agency for International Development National Institute for Agricultural Research	TC, RDM	[106]
September 2016	France	Working Group 38	DIAL, SOF, TC, RDM	[107]
October 2016 May 2017	Austria	ERA-NET Bioenergy	DIAL, TC, RDM	[108]
June 2017	Netherlands	Working Group 38	DIAL, SOF, TC, RDM	[107]

**Table 3:** Parameters of the regression lines (y = mx + q) obtained from the collected data as-is and excluding data obtained with wind speed lower than 2 m/s for the RDM techniques.

	DIAL	SOF	TC	RDM	RDM, U > 2 m/s
Slope (m)	0.868	1.468	1.263	−0.250	0.585
Intercept (q)	1.363	−1.379	−0.056	12.796	4.627
R^2^	0.8965	0.6527	0.8782	0.0147	0.1138

**Table 4:** Economic-logistic characteristics of the techniques used in the VERI study.

	TC	DIAL	RDM
Campaign duration (days)	1.5	1.5	1.5
Data analysis (hours)	40	50	100
Instrumentation cost (k€)	100	1500	70
Required personnel	2	3	2
Daily monitoring capacity (hectares/day)	>20	10 ÷ 15	>20

**Table 5:** Comparison of measured and calculated emission flow for the tank studied [57].

Tank	Tank Type	Content	Leaks Identified with OGI Camera	Emissions Estimated with API Algorithms [t/y]	Emissions Measured with TC Method [t/y]
T-105	EFRT ^1^	Crude oil	1	3	4
T-107	EFRT ^1^	Crude oil	4	4	35
T-108	EFRT ^1^	Crude oil	22	4	120
T-304	IFRT ^2^	Reforming gasoline	0	0.2	0
T-325	FRT ^3^	Fuel oil	0	1	0

^1^ EFRT = External Floating Roof Tank; ^2^ IFRT = Internal Floating Roof Tank; ^3^ FRT = Fixed Rood Tank.

## Abbreviations

The following abbreviations are used in this manuscript:

VOC	Volatile Organic Compound
DIAL	Differential Infrared Absorption Lidar
SOF	Solar Occultation Flux
TC	Tracer Correlation
OGI	Optical Gas Imaging
QOGI	Quantitative Optical Gas Imaging
RDM	Reverse Dispersion Modelling
BAT	Best Available Techniques
BREF	Best Available Techniques Reference
LDAR	Leak Detection and Repair
IR	Infrared Ray
UV	Ultra Violet
NPL	National Physical Laboratory
FTIR	Fourier Transform Infrared Ray
RPR	Release Precision Ratio
GWP	Global Warming Potential
PID	Photoionization Detector
FID	Flame Ionization Detector
ATEX	Atmosphères Explosives
API	America Petroleum Institute
SOCMI	Synthetic Organic Chemical Manufacturing Industry
EPA	Environmental Protection Agency
WG38	Working Group 38
VERI	Environnement Reasearch & Innovation
CRF	Controlled Release Facility
INERIS	Institut National de l’Environnement Industriel et des Risques
CFD	Computational Fluid Dynamic
SLAM	Safety Lagrangian Dispersion Model
